# Readiness for interprofessional learning among healthcare professional students

**DOI:** 10.5116/ijme.570d.7bd8

**Published:** 2016-05-12

**Authors:** Jaideep S. Talwalkar, Deborah B. Fahs, Gerald Kayingo, Risa Wong, Sangchoon Jeon, Linda Honan

**Affiliations:** 1 Departments of Internal Medicine and Pediatrics, Yale School of Medicine, CT, USA; 2 Division of Acute Care/Health Systems, Yale School of Nursing, CT, USA; 3 Betty Irene Moore School of Nursing, University of California Davis, Educ Bldg, Sacramento, CA, USA; 4 Department of Medicine, Massachusetts General Hospital, Boston, MA, USA

**Keywords:** Interprofessional education, readiness, students, attitudes

## Abstract

**Objectives:**

The purpose of this study was to investigate attitudes toward interprofessional learning among first year medical, nursing, and physician associate students at an American university at the start of their training.

**Methods:**

First year medical (n=101), nursing (n=81), and physician associate (n=35) students were invited to complete an anonymous online survey which included items related to demographic information and the Readiness for Interprofessional Learning Scale. Scores were compared by the general linear model and Duncan’s multiple range test while controlling for demographic differences.

**Results:**

All three groups scored in the high range, indicating readiness for shared learning. Female students, those with advanced degrees, and those with healthcare experience prior to enrolment in health professional school had significantly higher scores than their counterparts. After controlling for differences in demographic factors, nursing students scored significantly higher than physician associate and medical students (F_ (2,162)_ = 6.22, 0.0025).

**Conclusions:**

Health professions students demonstrated readiness for interprofessional learning early in their academic programs, however important differences in baseline readiness emerged. These findings suggest that educators consider baseline attitudes of students when designing interprofessional education curricula, and use caution when extrapolating data from other geographies or cultures.

## Introduction

American health education associations including the American Association of Colleges of Nursing and the Association of American Medical Colleges have collaborated to promote interprofessional education (IPE).^1^^,^^2^ In addition, the Liaison Committee on Medical Education^3^ recently adopted a new accreditation standard for medical schools in the United States requiring the incorporation of interprofessional curricular experiences into medical education, citing “the importance of IPE and interprofessional collaborative practice for ensuring improved patient outcomes and enhanced safety and quality of care.”

Recent qualitative studies^4^^,^^5^ have further detailed the importance of interprofessional practice and education. Improved patient outcomes have been linked to coordinated and collaborative practice,^6^^,^^7^while poor communication and lack of understanding of professional roles can result in patient care errors.^5^ Positive team experiences can decrease harmful stereotyping, improve understanding of roles and responsibilities, and boost confidence in one’s own ability to function on a team.^5^ 

There is an implied expectation that healthcare professionals will work together effectively as team members once in the workforce.^4^ Accordingly, it is no surprise that the need to incorporate IPE into training is widely accepted.^5^ Despite its importance, opportunities for learning with and about other healthcare professions is lacking in many training programs,^4^^,^^8^ and the incorporation of effective IPE into health professional training in the United States has much room for growth.^1^^,^^8^

A multitude of barriers to IPE implementation have been described, including structural and organizational conflicts related to program length and size, institutional support, geographic separation, faculty expertise, scheduling conflicts, and varied assessment methods and learning needs.^4^^,^^5^^,^^9^^,^^10^ However, variations in student attitudes towards IPE (e.g., prejudices, stereotypes) may be the biggest barrier of all.^5^^,^^9^^,^^10^^,^^11^ In a systematic review, Hammick and colleagues^9^ describe numerous IPE interventions with outcomes ranging from positive to neutral to ineffective. Although numerous factors impact the successful implementation of IPE, baseline student attitudes are among the most important factors influencing positive outcomes. These baseline attitudes can be based on a variety of factors including age, work experience, and gender.^9^ When considering the mixed success of past interventions, it becomes clear that simply bringing students from varied backgrounds and training programs together is insufficient to overcome pre-existing attitudinal barriers.^5^ Therefore, it is critical for educators to possess an understanding of student attitudes towards IPE prior to curricular design in order to take differences in values and beliefs into account.^11^

While the importance of IPE is gaining widespread acceptance in the United States, the bulk of the literature related to health professional student attitudes towards interprofessional collaboration at the start of training comes from other countries. Overall attitudes towards IPE have generally been favorable in these studies,^10^^,^^12^-^15^ yet findings have been inconsistent related to the predictive value of features such as professional program (e.g. medicine or nursing), age, gender, and prior healthcare experience, making it difficult to extrapolate this information to healthcare students in the United States. Less is known about readiness for and attitudes towards IPE among American healthcare professional students at the start of their training.^4^^,^^16^

The aim of this study was to determine baseline attitudes and perceptions toward IPE among first year medical, nursing, and physician associate (PA) students at an American university.

## Method

### Study participants

The study took place in three healthcare professional programs at Yale University which is located in the northeastern United States. Participants were first year students enrolled in the Yale School of Medicine (n=101), Yale School of Nursing (n=81), and Yale Physician Associate Program (n=35). Students in all three programs have completed their undergraduate (i.e., bachelor’s degree) training prior to matriculation at Yale.

Medical students complete a program lasting at least four years, resulting in an MD degree. Nursing students complete a three-year graduate-level program. Since entering students do not have a nursing background, they complete three years of fulltime study combining preparation in basic nursing during their first year (meeting the requirements for a certificate in nursing), and spend the remaining two years obtaining an advanced-practice degree (i.e., APRN or CNM). Physician associate students complete a two-year program resulting in a Master of Medical Science degree. Upon graduation, they sit for a national PA certification exam to become certified (PA-C). In all three programs, students may elect to spend additional time conducting research, or seeking other advanced degrees in conjunction with their primary training (e.g., MPH, PhD).

**Table 1:** Demographic characteristics of respondents by professional program (n=166)

Characteristic	Medical School	Physician Associate Program	School of Nursing	p
Respondents (%)	70 (69.3)	25 (80.1)	71 (87.7)	0.0110
Mean age in years ± Standard Deviation	23.8 ± 2.4	25.2 ± 2.8	26.3 ± 3.5	<0.0001
Female gender (%)	32 (45.7)	15 (62.5)	65 (91.5)	<0.0001
Advanced degree (%)	8 (11.4)	2 (8.3)	20 (28.2)	0.0145
Prior degree in humanities field (%)	16 (22.9)	1 (4.2)	35 (49.3)	<0.0001
Prior healthcare experience (%)	60 (85.7)	22 (88.0)	61 (87.1)	0.9478
Prior paid work in healthcare (%)	23 (32.9)	20 (80.0)	45 (63.4)	<0.0001
Prior healthcare work >2000 hours (%)	11 (15.7)	15 (60.0)	36 (50.7)	<0.0001

### Study instrument

First year students in the three professional programs were sent an anonymous online survey in January 2012. The survey included six items related to demographic information, as well as the 19-item Readiness for Interprofessional Learning Scale (RIPLS).

The RIPLS is a widely-utilized scale that measures the readiness of healthcare students for shared learning.^17^ Since its original description with three sub-scales,^11^ a newer four sub-scale model has been shown to be superior to the original.^17^ In a comparison of available instruments for measuring attitudes towards IPE, Thannhauser and colleagues^18^ identified the RIPLS as being among the most commonly used and psychometrically validated scales, and suitable for use in an academic context. Large samples have been used in a variety of psychometric validation studies of the RIPLS, specific items within the instrument, and its subscales.^17^^-^^20^ The RIPLS has been shown to be suitable to detect differences in attitudes towards IPE among students based on specific demographic characteristics, such as gender and year of training.^19^

The four sub-scales of the RIPLS are Teamwork/Collaboration, Positive Professional Identity, Negative Professional Identity, and Roles/Responsibilities. Teamwork/Collaboration consists of nine items measuring attitudes regarding the effect of cooperative learning.

**Table 2:** RIPLS* scores by selected demographic characteristics (n=166)

Demographic characteristic	Total RIPLS Mean ± SD	T&C^*^ Mean ± SD	NPI^*^ Mean ± SD	PPI^*^ Mean ± SD	R&R^*^ Mean ± SD
All respondents (n=166)	79.32 ± 8.04	38.89 ± 4.49	12.59 ± 1.91	16.11 ± 2.60	11.72 ± 2.35
Gender					
Female (n=112, 67.88%)	81.15 ± 6.48	39.71 ± 3.77	12.78 ± 1.68	16.32 ± 2.40	12.34 ± 2.16
Male (n=53, 32.12%)	75.63 ± 9.62	37.27 ± 5.37	12.15 ± 2.28	15.77 ± 2.91	10.43 ± 2.23
t_(df)_, p-value	t_(75)_=-3.80, 0.0003	t_(77)_=-2.98, 0.0038	t_(80)_=-1.79, 0.0767	t_(163)_=-1.27, 0.2054	t_(163)_=-5.25, <0.0001
Prior Healthcare Experience					
Yes (n=143, 86.67%)	79.17 ± 8.22	38.67 ± 4.56	12.59 ± 1.96	16.01 ± 2.60	11.90 ± 2.33
No (n=22, 13.33%)	79.98 ± 7.01	40.07 ± 3.82	12.64 ± 1.71	16.59 ± 2.54	10.68 ± 2.19
t_(df)_, p-value	t_(163)_=-0.44, 0.6631	t_(163)_=-1.37, 0.1739	t_(163)_=-0.10, 0.9180	t_(163)_=-0.98, 0.3305	t_(163)_=2.30, 0.0230
Advanced Degree					
Yes (n=30, 18.18%)	82.84 ± 6.12	40.77 ± 3.82	12.70 ± 1.91	16.87 ± 2.42	12.50 ± 2.03
No (n=135, 81.82%)	78.61 ± 8.21	38.52 ± 4.52	12.55 ± 1.92	15.98 ± 2.59	11.56 ± 2.39
t_(df)_, p-value	t_(163)_=2.66, 0.0086	t_(163)_=2.54, 0.0121	t_(163)_=0.38, 0.7022	t_(163)_=1.71, 0.0893	t_(163)_=2.00, 0.0471

*RIPLS: Readiness for Interprofessional Learning Scale; SD: Standard Deviation; T&C: Teamwork/Collaboration subscale; NPI: Negative Professional Identity subscale; PPI: Positive Professional Identity subscale; R&R: Roles/Responsibilities subscale

Positive Professional Identity consists of four items measuring the value placed on shared learning. Negative Professional Identity consists of three items that point to lack of value placed on cooperative learning. Roles/Responsibilities consists of three items that identify an unclear or distorted sense of what one’s professional roles might be. While higher scores on the RIPLS and its subscales indicate greater readiness for interprofessional learning, the items in the Negative Professional Identity and Roles/Responsibilities subscales are reverse coded.^17^ The RIPLS and its subscales are widely available in previously published work.^17^^,^^19^

Students received an in-class announcement about the survey from an investigator (JST, DBF or GK) who was a faculty member in their respective professional schools. Students received two reminders by email. The study was reviewed and granted exemption by the Yale University Institutional Review Board due to the educational nature of the project. All students invited to participate received descriptions of the study in both the in-class announcement and follow-up email reminders, with the understanding that participation was voluntary.

### Data analysis

Appropriate items on the Negative Professional Identity and Roles/Responsibilities subscales were reverse coded prior to calculations.^17^ Homogeneity in demographic characteristics among the three programs was assessed using chi-square test and analysis of variance (ANOVA). Mean scores on the RIPLS and its four subscales were compared by dichotomized demographic variables including gender, prior health care experience, and advanced degrees using t-test with a pooled standard deviation. In case of unequal variance between two groups, Satterthwaite approximation was used instead of pooled standard deviation. Due to unequal sample sizes across the programs, we used the general linear model instead of ANOVA to examine the difference in total RIPLS and subscale scores among the programs. Adjusted means of the RIPLS were compared after controlling for potential confounders including gender, prior health care experience, and advanced degree. In post hoc analysis, multiple comparisons between two programs were performed using Duncan’s multiple range test at a 0.05 significance level. All analyses were performed using SAS version 9.3.

## Results

Surveys were completed by 70 (69%) medical, 71 (88%) nursing, and 25 (71%) PA students. There were demographic differences among students (Table 1), some of which were associated with differences in scores on the RIPLS and its subscales.

Total RIPLS scores were statistically greater in female students (t_(__75)_=-3.80, p=0.0003) and those with advanced degrees (t_(163)_=2.66, p=0.0086) (Table 2), indicating more readiness for shared learning. In the analysis of RIPLS subscales, female students and those with advanced degrees demonstrated statistically higher scores on the Teamwork/Collaboration and Roles/Responsibilities subscales, while those with prior healthcare experience had statistically higher scores on the latter (Table 2). Age, bachelor’s degree in humanities, and prior paid job in healthcare had no significant impact on scores.

Overall, RIPLS scores were high among medical, nursing, and PA students, indicating strong readiness for IPE among all three groups. After controlling for differences in demographic factors, the three programs did not have equivalent scores in total RIPLS (F_ (2,162)_ = 6.22, p=0.0025), Teamwork/Collaboration (F_(__2,162) _= 4.62, p=0.0113), Positive Professional Identity (F_(2,162)_= 3.96, p=0.0210), and Roles/Responsibilities (F_(2,162)_=22.71, p<.0001) (Table 3). Duncan’s multiple range test at a 0.05 significance level revealed that nursing students scored significantly higher in total RIPLS and Teamwork/Collaboration compared to PA and medical students, and higher on Positive Professional Identity compared to PA students. Nursing and PA students had higher mean scores on the Roles/Responsibilities subscale compared to medical students (Table 3).

**Table 3:** RIPLS scores by professional program* (n=166)

Professional program	RIPLS† Mean ± SD†	T&C† Mean ± SD†	NPI† Mean ± SD†	PPI† Mean ± SD†	R&R† Mean ± SD†
Medical School	76.41 ± 8.92^b^	37.84 ± 4.89^b^	12.56 ± 2.13^a^	15.77 ± 2.67^a,b^	10.24 ± 2.24^b^
Physician Associate Program	76.84 ± 7.29^b^	36.84 ± 4.30^b^	12.48 ± 2.12^a^	14.92 ± 2.25^b^	12.60 ± 1.78^a^
School of Nursing	83.06 ± 5.56^a^	40.65 ± 3.44^a^	12.67 ± 1.61^a^	16.87 ± 2.45^a^	12.87 ± 1.76^a^
F _(df1,df2)_, p-value	F_(2,162)_=6.22, 0.0025	F_(2,162)_=4.62, 0.0113	F_(2,162)_=0.31, 0.7308	F_(2,162)_=3.96, 0.0210	F_(2,162)_=22.71, <0.0001

*Results controlled for demographic factors of gender, advanced degree, and prior healthcare experience. †RIPLS: Readiness for Interprofessional Learning Scale; SD: Standard Deviation; T&C: Teamwork and Collaboration subscale; NPI: Negative Professional Identity subscale; PPI: Positive Professional Identity subscale; R&R: Roles and Responsibilities subscale. a,b Groups with an identical superscripted letter are not statistically different by Duncan’s test at a significance level of 0.05.

## Discussion

Groups of students from three different healthcare professional schools at an American university demonstrated readiness for IPE when queried early in their academic programs. The high scores across learner groups were similar in magnitude to those reported by other educators,^12^^,^^13^ though some important distinctions emerged.

Nursing students demonstrated greater readiness for interprofessional learning than medical and PA students early in training, a finding similar to that reported in studies from Canada,^14^ New Zealand,^15^ and Sweden^21^ but different than reports from the United Kingdom,^13^ Singapore,^12^ the United States,^22^ and Iran.^23^ We were not surprised by the higher RIPLS scores among the nursing students in our study. The students in our nursing school seek advanced-practice degrees (i.e., Masters or PhD). Increasingly in the United States, advanced-practice nurses play important roles as organizers on healthcare teams composed of providers from various professional disciplines. Previous work suggests that entering nursing students think collaboratively, in contrast to medical students who think in individualistic terms.^15^ It is possible that students with an interest in team-based case are attracted to the nursing profession. In contrast, PA students learn in the medical model (as opposed to nursing), and the scores of these two groups of learners were similar - a finding that has not previously been reported to our knowledge.

 Similar to the findings of some previous studies, female students demonstrated more positive attitudes towards IPE than their male classmates.^10^^,^^13^^,^^14^^,^^21^ Coster and colleagues^13^ highlighted differences in learning styles between the genders that may account for women being more receptive to IPE. Specifically, women tend to emphasize listening, understanding, and trusting the views of others while learning. However, gender has not consistently been associated with differences in RIPLS scores across studies.^12^

While infrequently reported in published studies, a background of previous experience in healthcare prior to entering professional school has shown inconsistent associations with attitudes toward IPE.^13^^,^^21^ In our study, students with prior experience demonstrated higher scores on the Roles/Responsibilities subscale, suggesting a clearer sense of the professional roles of the different healthcare professions. Similarly, students with advanced degrees had higher RIPLS scores. Those with prior healthcare experience and advanced degrees likely have first-hand understanding of the contributions that various members of a healthcare or academic team can provide, explaining their higher scores. In contrast, simply being older or having been paid for work in healthcare prior to professional school did not translate to higher scores.

The disparate findings from studies performed worldwide suggest that differences in groups of learners may be attributable to cultural or educational background as well as differing selection criteria for healthcare professional schools. Accordingly, results from any one study might not be entirely applicable across schools or geographies, lending further support to the need to understand the attitudes of the local student community prior to IPE curricular design.^11^

By design, our study was limited in its scope to a single institution to inform our own curricular design. Additionally, response rates were highest among nursing students. Since the survey was anonymous, we had no mechanism to explore differences between respondents and non-respondents, though a plausible explanation for the difference in response rate might be nursing students’ increased readiness for shared learning as evidenced by their higher RIPLS scores.

## Conclusions

We found that healthcare professional students from three training programs within an American university demonstrated readiness for interprofessional learning early in their academic programs; however, important differences in baseline readiness emerged. These findings differ from those described among early learners outside the United States, suggesting that educators wishing to take baseline attitudes of students into account when designing interprofessional education curricula use caution when extrapolating data from other geographies or cultures.

### Conflict of Interest

The authors declare that they have no conflict of interest.
